# Immune and stromal remodeling underlies radiation-induced heart injury: insights from single-cell transcriptomics

**DOI:** 10.3389/fcvm.2026.1836858

**Published:** 2026-07-02

**Authors:** Xia Yan, JiaYi Zhao, QinYing Shi, Rui Yan, Sijin Li, Jianbo Song

**Affiliations:** 1 Third Hospital of Shanxi Medical University, Shanxi Bethune Hospital, Shanxi Academy of Medical Sciences, Tongji Shanxi Hospital, Taiyuan, Shanxi, China; 2 Shanxi Provincial Key Laboratory for Translational Nuclear Medicine and Precision Protection, Taiyuan, Shanxi, China; 3 Nursing College of Shanxi Medical University, Taiyuan, Shanxi, China; 4 Department of Nuclear Medicine, First Hospital of Shanxi Medical University, Shanxi Medical University, Taiyuan, Shanxi, China; 5 Shanxi Provincial People’s Hospital Affiliated to Shanxi Medical University, Taiyuan, Shanxi, China

**Keywords:** endothelium, fibroblast, immunity, radiation-induced heart injury, single-cell RNA-seq

## Abstract

**Background:**

Radiation-induced heart injury (RIHI) is a major late toxicity of thoracic radiotherapy, yet the cellular and molecular mechanisms driving its progression remain poorly defined.

**Methods:**

Single-cell RNA sequencing (scRNA-seq) was performed on rat hearts and matched peripheral blood mononuclear cells (PBMCs) 12 weeks after whole-heart irradiation (20 Gy) or sham control, profiling 38,941 cardiac cells across 15 types and 41,097 PBMCs across 9 types. Differential expression, pathway enrichment, pseudotime, and ligand–receptor interaction analyses were conducted. Key findings in endothelial cells and fibroblasts were validated by Western blotting and flow cytometry.

**Results:**

Major cardiac populations, including cardiomyocytes, endothelial cells (ECs), fibroblasts, neutrophils, macrophages, T cells, NK cells, and B cells, were defined in control and RIHI hearts. Following irradiation, ECs showed distinct subtype shifts with marked MHC-II upregulation, while fibroblasts exhibited iron accumulation, pro-inflammatory activation, and antigen-presenting properties. These stromal alterations coincided with myeloid activation (macrophage and IL-1β⁺ neutrophil programs) and T/NK cell polarization toward cytotoxic yet partially exhausted states, together with enhanced B-cell antigen presentation. Collectively, these findings delineate a stromal–immune cascade linking radiation injury to chronic cardiac inflammation.

**Conclusion:**

RIHI progresses through a stromal-immune cascade where EC and fibroblast immunogenic reprogramming initiates sustained myeloid and lymphoid activation, creating a pro-inflammatory cardiac microenvironment. These findings highlight non-hematopoietic antigen presentation as a therapeutic target in thoracic radiotherapy, particularly when combined with immune checkpoint inhibitors.

## Introduction

Radiation-induced heart injury (RIHI) is an increasingly recognized late complication of thoracic radiotherapy, with reported incidence rates ranging from 15% to over 30% depending on tumor type, radiation dose, and follow-up duration (1–3). In patients with lung, breast, and mediastinal malignancies, RIHI contributes substantially to cardiovascular morbidity and mortality, posing a critical challenge to long-term cancer survivorship (4, 5). Although endothelial cell (EC) damage is widely considered the initiating event, the progression of RIHI involves a complex interplay among fibroblasts, immune cells, and the extracellular matrix, ultimately leading to myocardial fibrosis, microvascular rarefaction, and contractile dysfunction (6–8).

Despite extensive research on RIHI pathophysiology, most mechanistic insights have been derived from tissue-level assays or bulk RNA sequencing, which obscure the heterogeneity of cellular responses and obscure the contributions of rare but functionally pivotal subpopulations (9, 10). Such approaches are insufficient to capture dynamic changes in immune activation, antigen presentation, and intercellular signaling within the injured cardiac microenvironment (11–13). For example, while EC activation and immune cell infiltration are recognized histologic hallmarks, the precise molecular programs driving their crosstalk and their consequences for disease progression remain poorly defined (14, 15).

Single-cell RNA sequencing (scRNA-seq) enables unbiased profiling of thousands of individual cells, providing high-resolution maps of cell identity, state, and lineage relationships (16, 17). It's broadly implied in decoding the molecular intricacies of diverse diseases, encompassing cancers, arthritis, lupus nephritis, asthma, pulmonary fibrosis, and radiation-induced lung injury (18–21). Recently, scRNA-seq is gaining traction in the investigation of heart diseases or injuries (22–24). Asp et al. revealed the comprehensive transcriptional landscape of cell types populating the embryonic heart at three developmental stages and that maps cell-type-specific gene expression to specific anatomical domains (25). Hua et al. delineated immune-cell programs in autoimmune myocarditis, implicating Hif1a-dependent inflammatory regulation (26). These studies highlight the potential of scRNA-seq to disentangle the immune-stromal complexity of RIHI.

Here, we applied single-cell RNA sequencing to paired cardiac tissue and peripheral blood mononuclear cells (PBMCs) from irradiated and control rats to generate a comprehensive single-cell atlas of RIHI. We profiled 38,941 cardiac cells (15 types) and 41,097 PBMCs (9 types), defined cell compositions and transcriptional states, and mapped antigen-presentation and chemokine networks across compartments. Endothelial subtypes (VECs/LECs) acquired immune-activated phenotypes with robust MHC-II and chemokine programs, while fibroblasts upregulated MHC-II pathways and exhibited iron accumulation with anti-ferroptosis adaptation. Myeloid and lymphoid compartments remodeled toward CD8⁺ cytotoxic activation with partial exhaustion and enhanced B-cell antigen presentation. Together, these data delineate a multicellular immune-metabolic circuit linking vascular-stromal “immunization” to sustained immune activation and nominate testable targets for cardio protection in thoracic radiotherapy, including RT-immunotherapy settings.

## Materials and methods

### Animal model and irradiation protocol

Sprague-Dawley rats were obtained from Shanxi Medical School. All experiments conformed to the guidelines from Directive 2010/63/EU and were approved by the local animal protection authorities (Shanxi Bethune Hospital, China).

Before irradiation, rats were anesthetized with 2% pentobarbital sodium at 0.2 mL/100 g and immobilized. After the accurate positioning of the irradiation area of rats with the simulator, a single dose of 20 Gy of 6.0 MV x-rays was delivered to a 1.5 cm × 1.5 cm area in the whole heart at a dose rate of 2.0 Gy/min. All other parts of the animal were shielded with a custom-made lead cover. The control rats (CN) were subjected to the same treatment except for irradiation. 12 weeks after radiation, the rats were euthanized by hemorrhagic shock to obtain fresh heart tissue and adequate blood samples. In addition, part of the heart was excised and immersed in 4% formaldehyde for at least 24 h before being embedded in paraffin. Sections (5 um) were cut and stained with hematoxylin/eosin (H&E) according to the standard method and observed with optical microscopy. Histological analysis of radiation-induced cardiac injury was independently assessed by two pathologists.

### Single-cell preparation and sequencing (scRNA-seq)

Single-cell suspensions at 1 × 10^5^ cells/mL in concentration in PBS (HyClone, Shanghai, China) were prepared and loaded onto microfluidic devices and scRNA-seq libraries were constructed according to Singleron GEXSCOPE® protocol by GEXSCOPE® Single-Cell RNA Library Kit (Singleron Biotechnologies) and Singleron Matrix® Automated single-cell processing system (Singleron Biotechnologies). Scanpy v1.8.1 was used for quality control, dimensionality reduction, and clustering under Python 3.7. The raw count matrix was normalized by total counts per cell and logarithmically transformed into a normalized data matrix. The top 2,000 variable genes were selected by setting flavor = “seurat”. Principal Component Analysis (PCA) was performed on the scaled variable gene matrix, and the top 20 principal components were used for clustering and dimensional reduction. Cells were separated into 28 (Heart)/24(PBMC) clusters by using the Louvain algorithm and setting the resolution parameter at 1.2. Cell clusters were visualized by using Uniform Manifold Approximation and Projection (UMAP). A resolution of 1.2 was selected because it preserved major lineage identities while providing sufficient separation of biologically distinct subpopulations without excessive cluster fragmentation.

### Differentially expressed genes (DEGs) analysis (Scanpy)

To identify differentially expressed genes (DEGs), we used scanpy. tl. rank_genes_groups() function based on the Wilcoxon rank sum test with default parameters and selected the genes expressed in more than 10% of the cells in either of the compared groups of cells and with an average log (Fold Change) value greater than 0.25 as DEGs. Adjusted *p*-value was calculated by Benjamini–Hochberg correction and the value 0.05 was used as the criterion to evaluate the statistical significance.

### Pathway enrichment analysis

To investigate the potential functions of subcluster cells, Gene Ontology (GO) and Kyoto Encyclopedia of Genes and Genomes (KEGG) analysis were used with the “clusterProfiler” R package v 3.16.1. Pathways with a *p*-adj value less than 0.05 were considered as significantly enriched.

### UCell gene set scoring

Gene set scoring was performed using the R package UCell v 1.1.0. UCell scores are based on the Mann–Whitney *U* statistic by ranking query genes in order of their expression levels in individual cells. Because UCell is a rank-based scoring method, it is suitable to be used in large datasets containing multiple samples and batches.

### Pseudotime trajectory analysis: Monocle2

Cell differentiation trajectory of monocyte subtypes was reconstructed with the Monocle2 v 2.10.0. For constructing the trajectory, the top 2,000 highly variable genes were selected by Seurat (v3.1.2) FindVairableFeatures(), and dimension-reduction was performed by DDRTree(). The trajectory was visualized by the plot_cell_trajectory() function in Monocle2.

### Transcription factor regulatory network analysis (pySCENIC)

The transcription factor network was constructed by pyscenic (v0.11.0) using scRNA expression matrix and transcription factors in AnimalTFDB. First, GRNBoost2 predicted a regulatory network based on the co-expression of regulators and targets. CisTarget was then applied to exclude indirect targets and to search transcription factor binding motifs. After that, AUCell was used for regulon activity quantification for every cell. Cluster-specific TF regulons were identified according to Regulon Specificity Scores (RSS), and the activity of these TF regulons was visualized in heatmaps.

### Western blot and flow cytometry

After 48 h of irradiation (24Gy), endothelial cells were lysed in RIPA buffer containing protease and phosphatase inhibitors. Equal amounts of protein (20 µg) were resolved by 10%–12% SDS–PAGE, transferred onto PVDF membranes, blocked with 5% BSA, and incubated overnight at 4 °C with primary antibodies against MHC-II (RT1B, clone OX-6, 1:1000, Abcam). HRP-conjugated secondary antibodies were used for detection, and signals were visualized with enhanced chemiluminescence (ECL). Densitometric quantification was performed using ImageJ, normalized to GAPDH (1:5000, CST).

Fibroblast cells were harvested, washed with PBS, and incubated with FerroOrange (1 µM, Dojindo, Japan) in serum-free medium at 37 °C for 30 min in the dark. After washing with PBS, fluorescence intensity was detected on a flow cytometer (excitation/emission: 543/580 nm, PE channel). Data were analyzed using FlowJo software to quantify intracellular Fe²⁺ levels.

## Results

### Cell atlas of RIHI heart and PBMC

**Figure 1:**
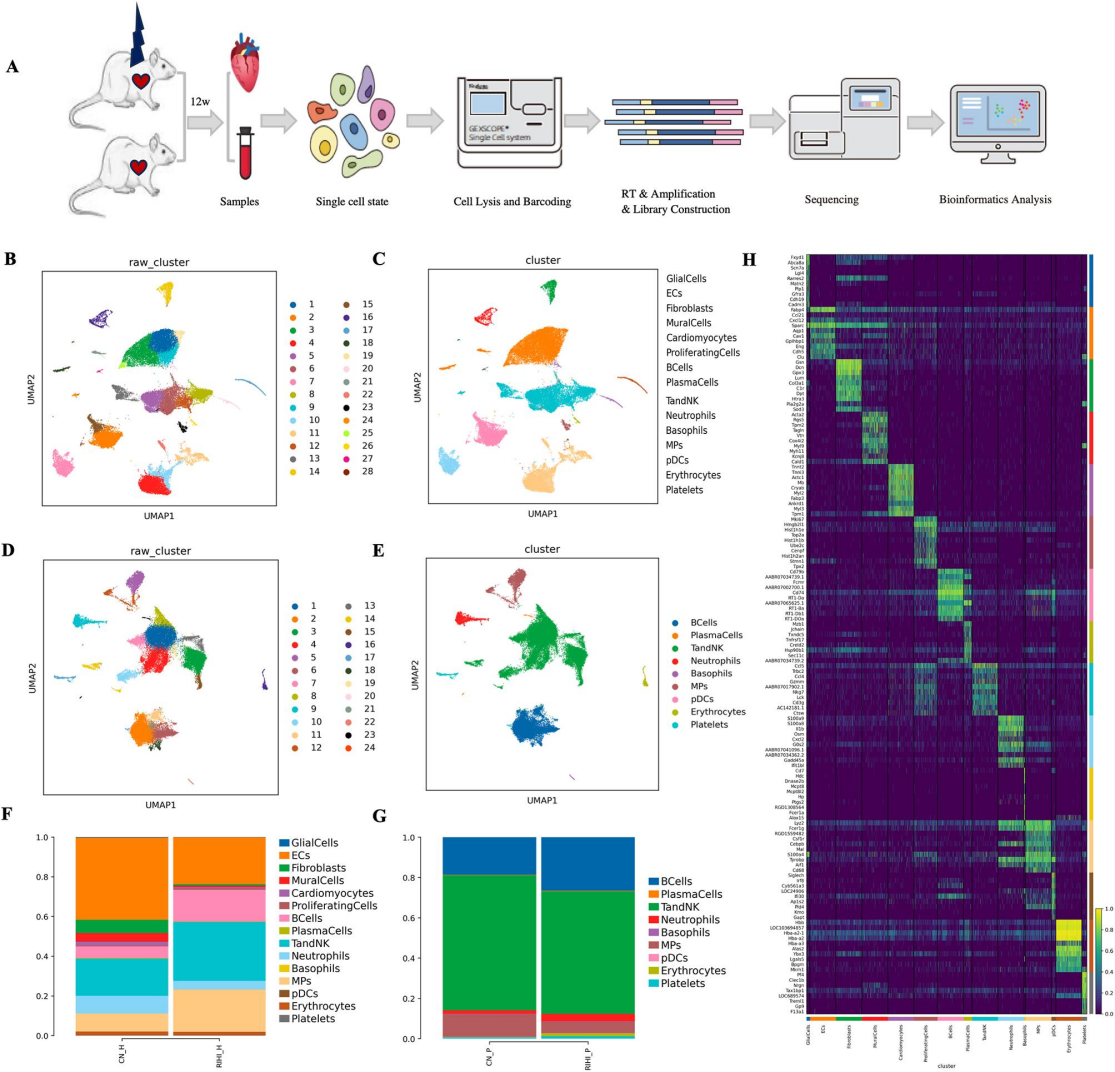
The single-cell landscape of rat heart and PBMC before and after irradiation. **(A)** Workflow of experiment design; **(B,C)** UMAP for all clusters and cell types of the heart (*n* = 38,941 cells); **(D,E)** UMAP for all cluster and cell types of PBMC (*n* = 41,097 cells); **(F)** The relative proportion of each cell cluster between groups is indicated. CN_H: the heart tissue samples from the control rat, one simple; RIHI_H: the heart tissue samples from the irradiated rat, two replicates; **(G)** The relative proportion of each cell cluster across 2 groups is indicated. CN_P: the PBMC samples from the control rat, one sample; RIHI_P: the PBMC samples from the irradiated rat, two replicates; **(H)** Heatmap showing the differentially expressed genes of cell types ordered by hierarchical clustering.

To comprehensively profile the transcriptional response to irradiation, we performed scRNA-seq on unsorted cells from rat hearts and matched PBMCs 12 weeks after whole-heart irradiation or control, a time point selected based on H&E evidence of cardiac injury (Figure 1A, Supplementary Figures S1A,B). After stringent quality control, 38,941 high-quality cardiac single-cell transcriptomes and 41,097 PBMC transcriptomes were retained for analysis.

In cardiac tissue, unsupervised clustering resolved 28 clusters, including cardiomyocytes (cluster 20), immune cells (clusters 2, 4–8, 10, 11, 13, 15, 22–24, 26), endothelial cells (clusters 1, 3, 9, 19, 21, 25), and other stromal populations (clusters 12, 14, 16–18) (Figure 1B). Using canonical markers and differentially expressed genes, we annotated 15 major lineages: Endothelial cells (“ECs”, Cdh5 and Flt1), Fibroblasts (“Fibroblasts”, Col1a1 and Dcn), Mural cells (“Mural Cells”, Acta2 and Rgs5), Cardiomyocytes (“Cardiomyocytes”, Myh6 and Tnnt2), Proliferating cells (“Proliferating Cells”, Mki67 and Top2a), B cells (“B Cells”, Cd19 and Cd79b), Plasma cells (“Plasma Cells”, Jchain and Mzb1), T and NK cells (“T and NK”, Cd3), Neutrophils (“Neutrophils”, S100a8/a9), Basophils (“Basophils”, Srgn and Ifitm1), Mononuclear phagocytes (“MPs”, Lyz2 and Cd68), Plasmacytoid dendritic cells (“pDCs”, Siglech and Irf7), Erythrocytes (“Erythrocytes”, Hba-a2 and Slc4a1), Platelets (“Platelets”, Pf4 and Ppbp), Glial cells (“Glial Cell”, Plp1 and Scn7a) (Figures 1C,H, Supplementary Figure S1C). In PBMCs, analogous analysis identified 24 clusters grouped into nine phenotypic classes: B cells, plasma cells, T/NK cells, neutrophils, basophils, mononuclear phagocytes, plasmacytoid dendritic cells, erythrocytes, and platelets (Figures 1D,E). The proportions of each cell type are reported in the Supplementary Tables S1 and S2.

Comparative composition analysis revealed pronounced immunological remodeling in the heart after irradiation: T/NK cells, B cells, and mononuclear phagocytes increased, whereas endothelial cells, fibroblasts, cardiomyocytes, and neutrophils decreased (Figure 1F). In contrast, PBMCs displayed a reciprocal pattern, with B cells and neutrophils increasing and T/NK cells and mononuclear phagocytes decreasing (Figure 1G), consistent with compartmentalized redistribution between tissue and blood.

### Radiation-induced antigen-presenting activation in cardiac endothelium

**Figure 2:**
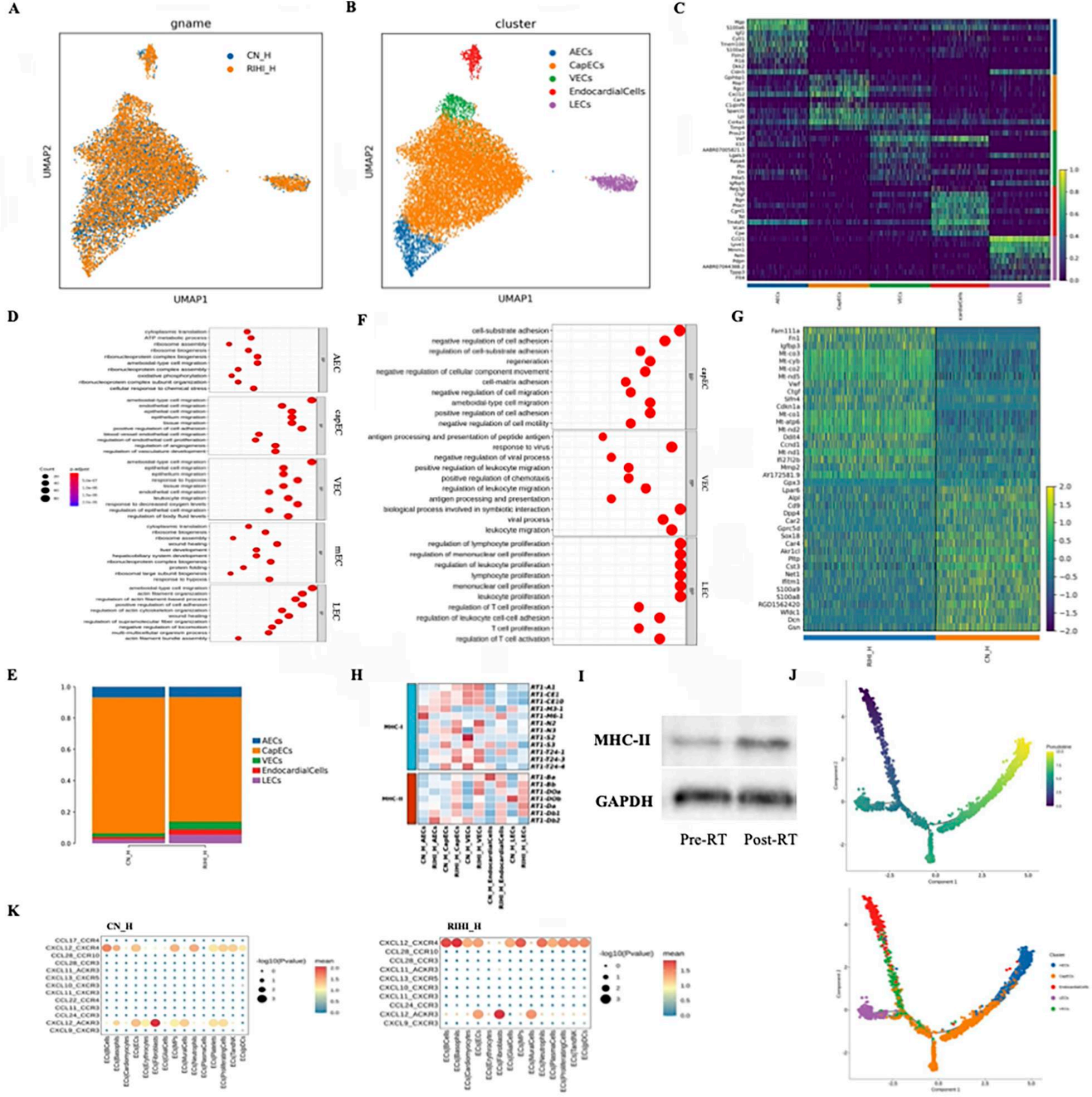
Endothelial cell profiling and transcriptional remodeling after irradiation. **(A,B)** UMAP for cell types of RIHI and control heart, including AECs, CapECs, VECs, Endocardial Cells, and LECs; **(C)** Heatmap of marker genes defining endothelial subsets; **(D)** Representative top 10 GO terms enriched in different EC types; **(E)** The relative proportion of each cell cluster between groups; **(F)** The representative top 10 GO terms enriched in different EC types in RIHI samples; **(G)** Heatmap showing upregulated genes in CapECs post-irradiation; **(H)** Heatmap of MHC-I and MHC-II pathway genes across endothelial subclusters pre- and post-irradiation; **(I)** Western blot validation of MHC-II in endothelial cells before and after irradiation; **(J)** Pseudotime trajectory analysis of endothelial cells; **(K)** Ligand–receptor interaction analysis highlighting chemokine-mediated communication patterns in control (CN) and irradiated (RIHI) conditions.

The prevailing perspective suggests that endothelial dysfunction plays a pivotal role in the pathogenesis of RIHI. Through comprehensive single-cell profiling, a total of 10,948 endothelial cells were discerned and classified into five clusters, including: arterial (AEC), capillary (CapEC), venous (VEC), lymphatic (LEC), and endocardial subsets (Figures 2A,B). Baseline gene ontology highlighted migration programs in CapECs (e.g., Rgcc, Sparcl1), hypoxia responses in VECs (e.g., Eln, Icam1), actin cytoskeleton organization in LECs (e.g., Ccl21, Lyve1, Flt4), ribosome in AECs, and ER protein processing in endocardial cells (Figures 2C,D).

Radiation exposure reshaped the endothelial compartment, with CapECs decreasing and VECs/LECs expanding (Figure 2E). In CapECs, mitochondrial genes (Mt-co1–3, Mt-cyb, Mt-nd1/2/5) and ECM-associated transcripts (Fn1, Vwf, Spp1) were upregulated, consistent with impaired oxidative phosphorylation and enhanced matrix remodeling (Figures 2F,G). VECs exhibited an immune-activated phenotype, with induction of antigen-presentation and chemotaxis programs (e.g., Cd74, RT1 family, Ccl4, Ccl5) (Figure 2F, Supplementary Figure S2A), while LECs upregulated MHC-II-related and lymphocyte regulatory genes (RT1-Db1, Cd74, Coro1a) (Figure 2F, Supplementary Figure S2B). Motivated by these features, we profiled MHC pathway genes across EC subtypes: MHC-II genes (RT1-Db1, RT1-DOb, RT1-Bb) were robustly elevated in VECs, LECs, and endocardial cells after irradiation, whereas MHC-I genes (e.g., RT1-N3, RT1-S2) showed moderate increases mainly in VECs and CapECs (Figure 2H). The WB of irradiated ECs confirmed an increase in MHC-II protein, compared with the control group (Figure 2I).

Trajectory inference reconstructed a continuum from endocardial/VEC early states through a CapEC transitional hub to an AEC end-state, with a side branch toward LECs. Along pseudotime, homeostatic programs (Apoe, Reg3b, Bgn) declined, whereas ECM-remodeling and arterial-identity genes (Mgp, Cst3, Tmem100, Sox17) progressively increased, concordant with the matrix-activated phenotype after irradiation. A distinct LEC trajectory peaked with Ccl21, aligning with the induction of MHC-II and lymphocyte-regulatory pathways and suggesting enhanced immune-cell recruitment (Supplementary Figures S2C,D). EC-centered chemokine maps show a broadened CXCL12–CXCR4 network after irradiation, from a few partners pre-RT to most immune/stromal compartments, with reinforced CXCL12-ACKR3 auto/paracrine loops (Figure 2J).

### Fibroblasts acquire immunoregulatory feature with ferroptosis adaptation after irradiation

**Figure 3:**
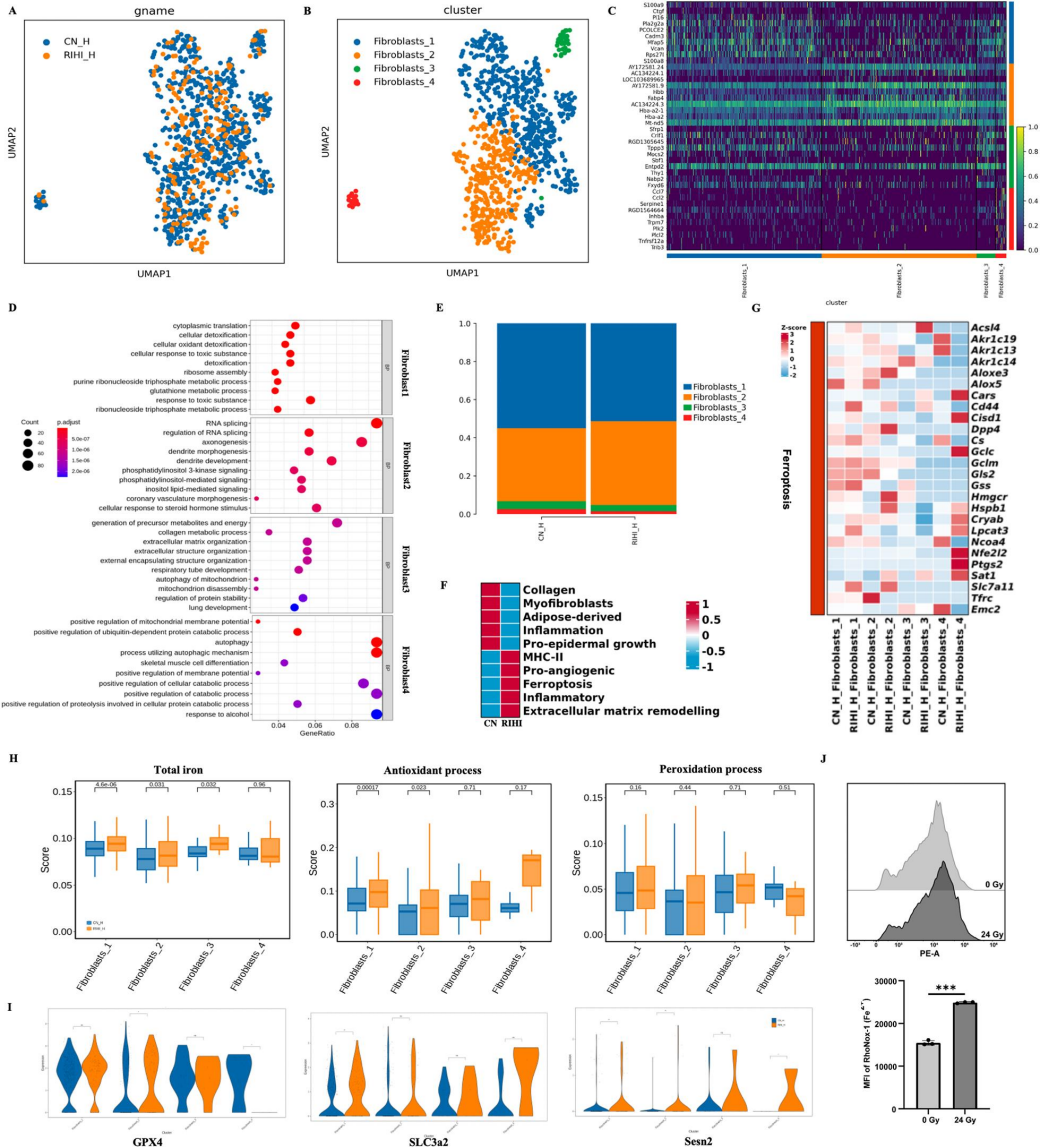
Fibroblast remodeling and ferroptosis-related signatures after irradiation. **(A,B)** UMAP for cell types of RIHI and control heart, fibroblasts 1–4 (*n* = 919 cells); **(C)** Heatmap of marker genes defining fibroblast subsets; **(D)** Dotplot showing the representative top 10 GO terms enriched in different fibroblast types; **(E)** The relative proportion of each cell cluster between groups; **(F)** Module scoring of signature genes in all fibroblasts pre- and post-irradiation; **(G)** Heatmap of ferroptosis-related genes across fibroblast subclusters pre- and post-irradiation; **(H)** Boxplot of total iron, antioxidant process, and peroxidation process in fibroblast subclusters; **(I)** Violin plots showing the expression of Gpx4, SLC3a2, and Sesn2 in fibroblast subclusters pre-and post-irradiation. The *p*-value corresponds to the paired *t*-test; **(J)** Flow cytometric analysis of intracellular Fe^2+^ levels in endothelial cells before and after irradiation.

We next examined the population of fibroblasts, defining four subclusters, including: fibroblast_1 (enriched for nucleotide metabolism), fibroblast_2 (RNA splicing), fibroblast_3 (extracellular matrix organization), and fibroblast_4 (cellular catabolic processes) (Figures 3A–D).

After irradiation, the proportion of fibroblast_2 increased, while fibroblast_1/3/4 decreased (Figure 3E). Gene-set profiling showed upregulation of antigen processing/presentation, inflammatory, and ECM programs in RIHI fibroblasts (Figure 3F). Subcluster analysis showed enrichment of ferroptosis-related programs in fibroblast_1 and fibroblast_2 (Figure 3G). Further analysis indicated that while total intracellular iron levels were elevated in these subtypes, labile iron pools remained unchanged. Moreover, transcriptional signatures indicated an increase in antioxidant defenses rather than lipid peroxidation, with an increase of GPX4, Sesn2, and SLC3a2, factors implicated in the ferroptosis process (Figures 3H,I). In addition, flow-cytometric iron probes demonstrated increased intracellular iron after irradiation, in line with the transcriptional rise in total-iron signatures (Figure 3J). These findings suggest that fibroblasts, particularly fibroblast_2, may adopt an immune-activated yet ferroptosis-resistant phenotype after irradiation.

Trajectory inference ordered fibroblasts along a single continuum with a minor spur, placing fibroblast_1/2 in early–intermediate states and fibroblast_3/4 at late pseudotime. Early injury programs (Apoe, S100a8/S100a9, Clu) declined, while ECM/myofibroblast modules (Ctgf, Fbn1, Vcan, Mfap5, ECM1, Pla2g2a, Uap1) progressively increased, indicating a stepwise transition toward matrix remodeling. A late Pi16^high^ endpoint and enrichment of stress-adaptive signals align with an immune-activated yet ferroptosis-guarded phenotype in fibroblasts, particularly fibroblast_2 (Supplementary Figures S3A–D).

### Irradiation shifts macrophages to Fcna-high/MHC-I-enriched states

**Figure 4:**
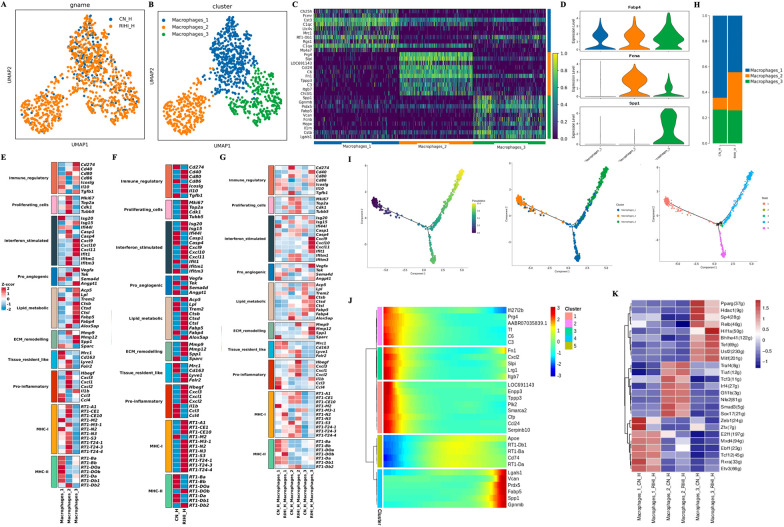
Macrophage remodeling and trajectory dynamics after irradiation. **(A,B)** UMAP for cell types of RIHI and control heart, macrophages 1_3 (990 cells); **(C)** Heatmap of canonical markers for macrophage subtype identification; **(D)** Violin plots showing the expression of Fabp4, Fcna, and Spp1 in macrophages 1_3; **(E)** Heatmap showing the scaled expression level of signature genes in macrophages 1_3; **(F)** Heatmap showing the relative change in the expression level of signature genes in all macrophages pre- and post-irradiation. **(G)** Heatmap showing the relative change in the expression level of signature genes in each macrophage subcluster pre- and post-irradiation; **(H)** The relative proportion of each cell cluster between groups; **(I)** Unsupervised transcriptional trajectory of macrophages 1_3. The color from heavy to light represents development from beginning to end; **(J)** Heatmap of pseudotime-associated genes; **(K)** Heatmap of transcription factors driving macrophage state transitions. The color key from blue to red indicates relative expression levels from low to high.

Traditional classifications of macrophage activation states, such as M1 classical activation or M2 alternative activation, have proven insufficient in capturing the nuanced diversity of these cells, as revealed by advancing detection technologies. In our study, 990 cardiac macrophages were identified and classified into 3 heterogeneous subgroups (Figures 4A,B). Subtype-resolved heatmaps revealed clear functional diversity among the three macrophage clusters: Macrophage_1 (Spp1^low^, Fcn^low^, Fabp4^+^) was the predominant subtype and expressed high levels of complement components (C1qa, C1qc) and MHC-II genes, along with the tissue-residency markers Cd163 and Mrc1, consistent with an anti-inflammatory, M2-like phenotype. Macrophage_2 (Spp1^low^, Fcn^high^, Fabp4⁺) displayed elevated expression of proliferation and lymphatic markers (Mki67, Lyve1), along with upregulation of MHC-I genes, suggesting a role in antigen presentation and immune activation. Macrophage_3 (Spp1^high^, Fcn^low^, Fabp4⁺) exhibited gene signatures associated with lipid metabolism (Fabp5), extracellular matrix remodeling (Mmp12), and interferon-stimulated genes (Cxcl9-11), reflecting a metabolically active and inflammatory state (Figures 4C–E).

After irradiation, macrophage_1 decreased, and macrophage_2 increased in proportion (Figure 4H). Across all macrophages, post-irradiation changes included higher inflammatory and activation signatures, such as upregulation of antigen-presentation machinery, with notable upregulation of MHC class I genes (RT1-A1) and immune-stimulatory molecules (Cd86, Cd40) in irradiated macrophages (Figure 4F). At the subtype level, macrophage_2 maintained strong pro-inflammatory features and displayed post-irradiation elevation of MHC-I related/antigen-processing signatures, whereas macrophage_3 showed the most pronounced induction of lipid-metabolic and interferon programs (Figure 4G). These patterns are consistent with enhanced antigen handling together with state-specific inflammatory and metabolic adaptation in RIHI.

Pseudotime arranged macrophages from an early Macrophages_2 state, bifurcated into two endpoints. One branch (mainly Macrophages_1) gained an MHC-II antigen-presenting program (RT1-Db1/Ba/Da, Cd74) with rising Apoe, while the other (predominant Macrophages_3) acquired a SPP1^+^/GPNMB^+^ wound-healing/pro-fibrotic phenotype, co-expressing Vcan, Lgals1, Fabp5, and Prdx5 (Figures 4I,J). Transcriptional regulon analysis via SCENIC highlighted TRAF4 and TIA1 as key regulators of Macrophage_2, and HIF1A of Macrophage_3 (Figure 4K).

### NFATC1 program is concomitantly engaged in neutrophil activation after irradiation

**Figure 5:**
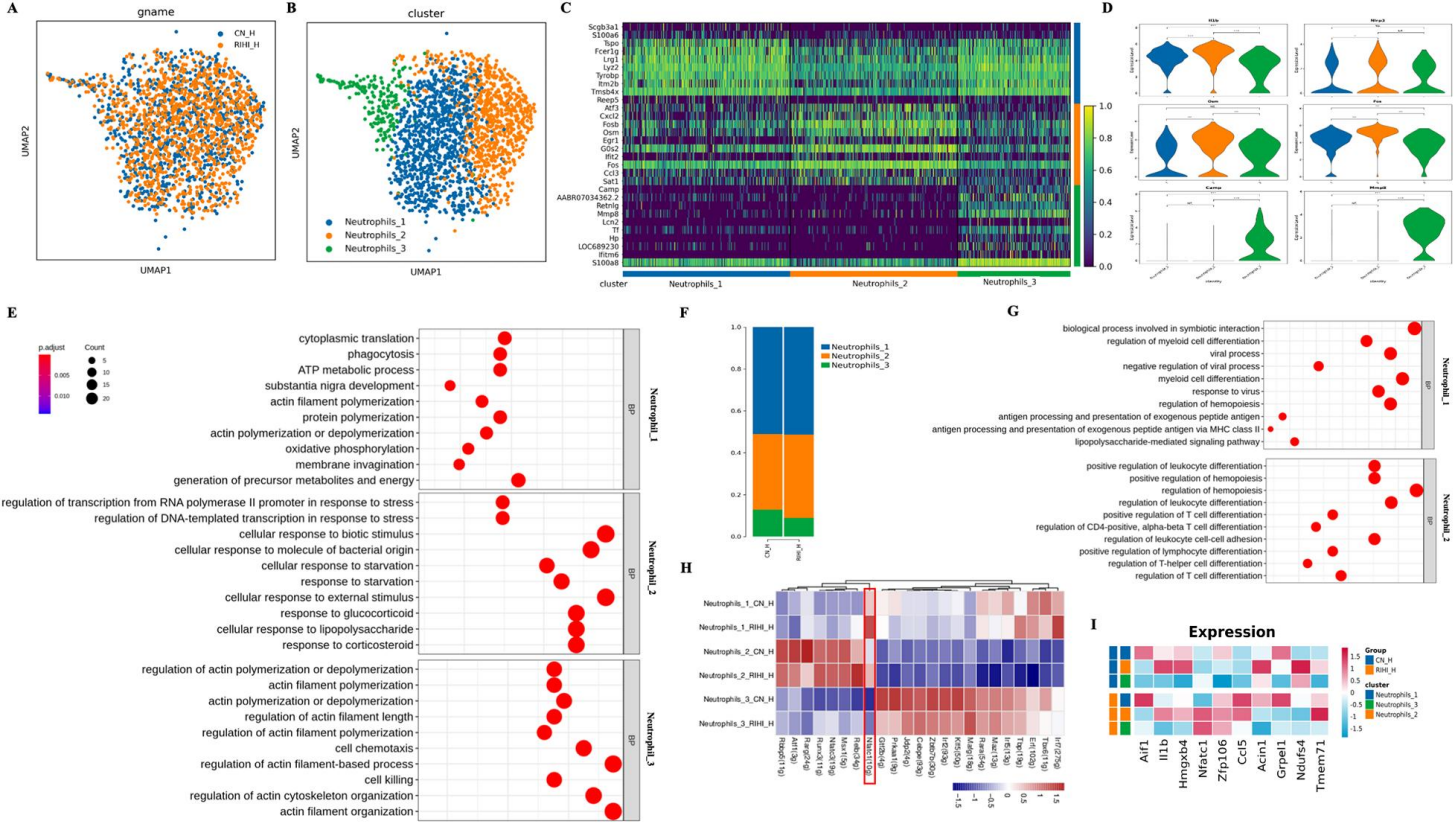
Neutrophil remodeling and functional programs after irradiation. **(A,B)** UMAP for cell types of RIHI and control heart, neutrophils1_3 (*n* = 1,904 cells); **(C)** Heatmap of marker genes defining neutrophil subsets; **(D)** Violin plots showing representative marker genes across neutrophil subclusters; **(E)** The representative top 10 GO terms enriched in different neutrophil cell types; **(F)** The relative proportion of each cell cluster between groups; **(G)** The representative top 10 GO terms enriched in neutrophil_1 and neutrophil_2 in RIHI samples; **(H)** The heatmap exhibits the average regulon activities of transcription factors in different neutrophil cell types; **(I)** The expression of Nfatc1 target genes. The color key from blue to red indicates relative expression levels from low to high.

Neutrophils are pivotal effectors of innate immunity and act as key amplifiers of inflammatory responses in RIHI. A total of 1904 neutrophils were classified into three distinct clusters (Figures 5A,B). Violin plot analysis showed that IL-1β was expressed across all neutrophil populations, with cluster 2 exhibiting the highest expression. This IL-1β enrichment in cluster 2 coincided with NLRP3 expression, consistent with the known role of the NLRP3 inflammasome in IL-1β maturation (27). All clusters expressed the calcium-binding proteins S100a6 and S100a8 (Figures 5C,D). Cluster 1 (e.g., Scgb3a1, Tspo, Itm2b) showed programs related to cytoplasmic translation, phagocytosis, and ATP metabolism, indicating a metabolically active state. Cluster 2 exhibited Fos/OSM/Cxcl2 upregulation, consistent with transcriptional activation and inflammatory signaling. Cluster 3 shared elements with cluster 1 (Tspo, Fcer1g) but also expressed Camp, Retnlg, Mmp8, suggestive of tissue repair/clearance functions (Figures 5C,E).

After irradiation, the proportion of cluster 2 modestly increased, while cluster 3 declined (Figure 5F). Subcluster analysis revealed enhanced antigen processing and presentation in cluster 1 and increased leukocyte activation signatures in cluster 2 (Figure 5G). KEGG analysis further linked neutrophil transcriptional changes to T-helper differentiation (Th1/Th2/Th17) and the PD-1/PD-L1 axis (Supplementary Figures S4A–C).

Regulon analysis indicates that NFATC1 is transcriptionally active in neutrophil clusters 1 and 2 (Figure 5I). Consistently, NFATC1 target modules tied to leukocyte activation and chemotaxis (e.g., Ccl5) are upregulated. These changes parallel GO enrichments, antigen processing/presentation in cluster 1, and positive regulation of leukocyte activation in cluster 2, supporting a model in which, after irradiation, neutrophils shift from resting to effector-like states via an NFATC1-linked program.

### T/NK compartment shifts toward cytotoxic programs with metabolic constraint

**Figure 6:**
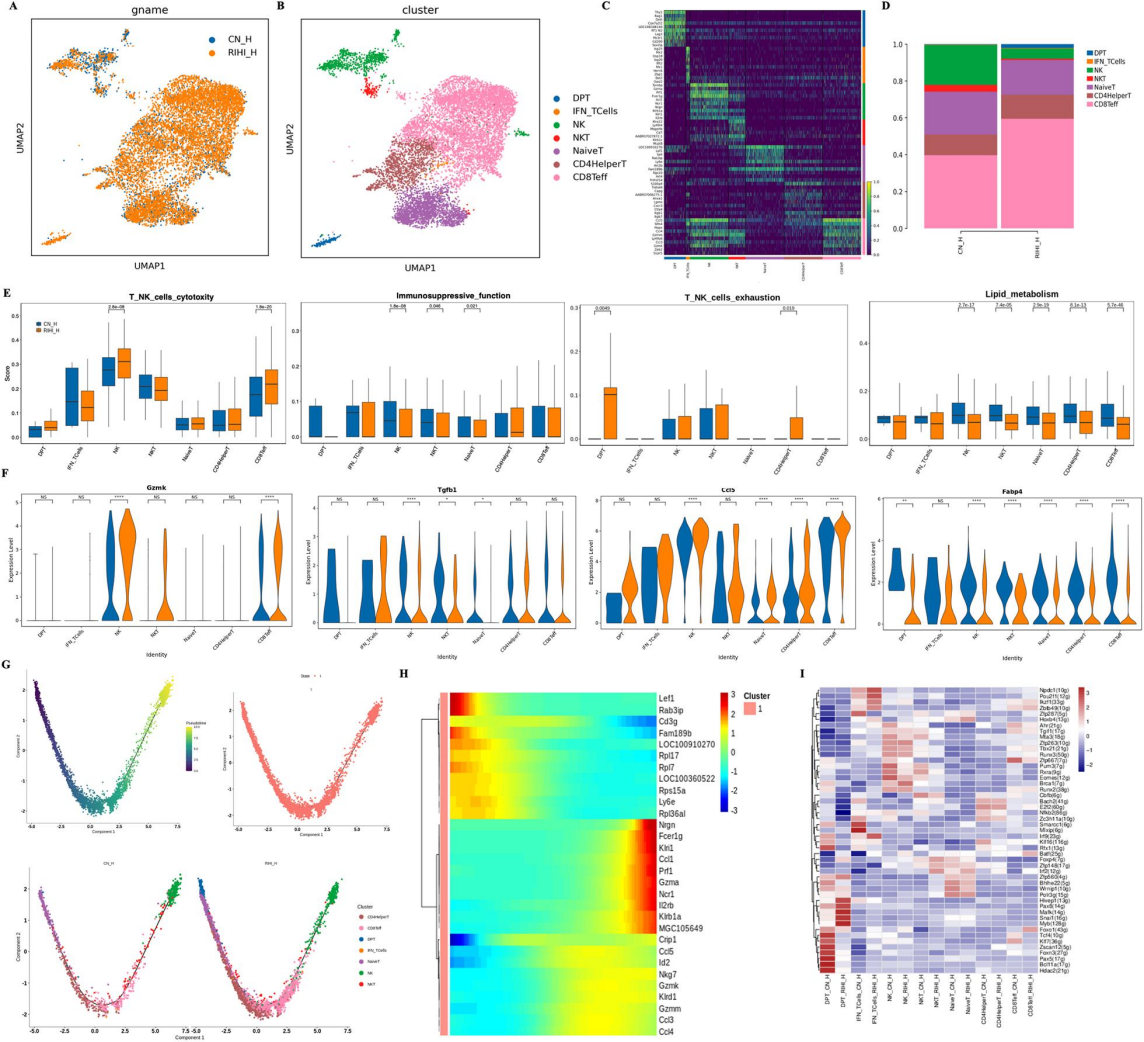
T and NK cell remodeling and functional states after irradiation. **(A,B)** UMAP for cell types of RIHI and control heart, including DPT, IFN_T, NK, NKT, NaiveT, CD4HelperT, and CD8Teff cells (*n* = 10,174 cells); **(C)** Heatmap of marker genes for T and NK cell subtype identification; **(D)** The relative proportion of each cell cluster between groups; **(E)** Boxplot showing the changes in the average expression of signature genesets in T and NK subclusters pre- and post-irradiation; **(F)** Violin plots showing the expression of Gzmk, Tgfb1, Ccl5, and Fabp4 in T and NK subclusters pre- and post-irradiation. The *p*-value corresponds to the paired *t*-test; **(G)** Trajectory of T and NK cells. The color from heavy to light represents development from beginning to end; **(H)** Heatmap of pseudotime-associated genes indicating state-specific transcriptional programs; **(I)** Heatmap of transcription factors and regulators enriched in distinct T/NK cell subsets. The color key from blue to red indicates relative expression levels from low to high.

T and NK cells are central to immune surveillance and tissue inflammation, and their activation state critically shapes RIHI pathophysiology. We profiled 10,174 cardiac T/NK cells and resolved seven transcriptional states: NK cells (Ncr1^+^, Nkg7^+^, Gzma^+^), NK T cells (Klrb1c^+^, Cd8a^+^), double-positive T cells (DPT; CD4^+^, CD8a^+^, RAG1^+^), interferon-responsive T cells (IFN_T; Ifit3^+^, Isg15^+^), naïve T cells (Sell^+^, Lef1^+^, Tcf7^+^), CD4^+^ T helper cells (Tnfrsf4^+^, Cd3g^+^, Gata3^+^), and CD8^+^ effector T cells (Cd8a^+^, Ccl4^+^, Ccl5^+^) (Figures 6A–C). In PBMCs, only naïve T cells, regulatory T cells, CD8^+^ effector T cells, and NK cells were detectable, indicating compartment-specific T/NK heterogeneity (Supplementary Figures S4D–G).

After irradiation, naïve T cells declined, whereas CD8^+^ effector T cells and IFN_T cells expanded in the heart (Figure 6D). In peripheral blood, the CD4:CD8 ratio increased (Supplementary Figure S5D). These changes are consistent with a shift from resting to activated/effector states within the cardiac compartment. Consistent with the gene-set scores, cytotoxic programs peaked in NK and CD8^+^ effector T cells, mirrored at the single-gene level by higher Gzmk. By contrast, exhaustion scores increased predominantly in DPT and CD4^+^ helper T cells, with only modest changes in NK/CD8^+^ effectors, indicating that cytotoxic enhancement and exhaustion are partly decoupled across lineages. Chemokine competence was reinforced by increased Ccl5 across multiple T-cell subsets, whereas immunosuppressive signaling declined, reflected by lower Tgfb1. In parallel, reduced Fabp4 suggested a shift in lipid-metabolic capacity (Figures 6E,F).

Pseudotime analysis positioned DPT cells at the trajectory origin, progressing toward CD8^+^ effector and NK termini (Figure 6G). Along this path, Ccl3/Ccl4/Ccl5 increased, and GZMA/B/K peaked in terminal NK states (Figure 6H), indicating coordinated acquisition of chemokine and cytotoxic programs during maturation. The regulon heatmap mirrored this subset specificity: Myb/Snai1/Mafk/Hivep1 regulons predominated in DPT/naïve/IFN_T compartments, whereas Foxo1-associated regulons were more prominent in CD8^+^ effector/NK compartments, aligning with the observed shift from resting to effector states (Figure 6I).

### Naïve B-cell dominance with enhanced antigen-presentation capacity after irradiation

**Figure 7:**
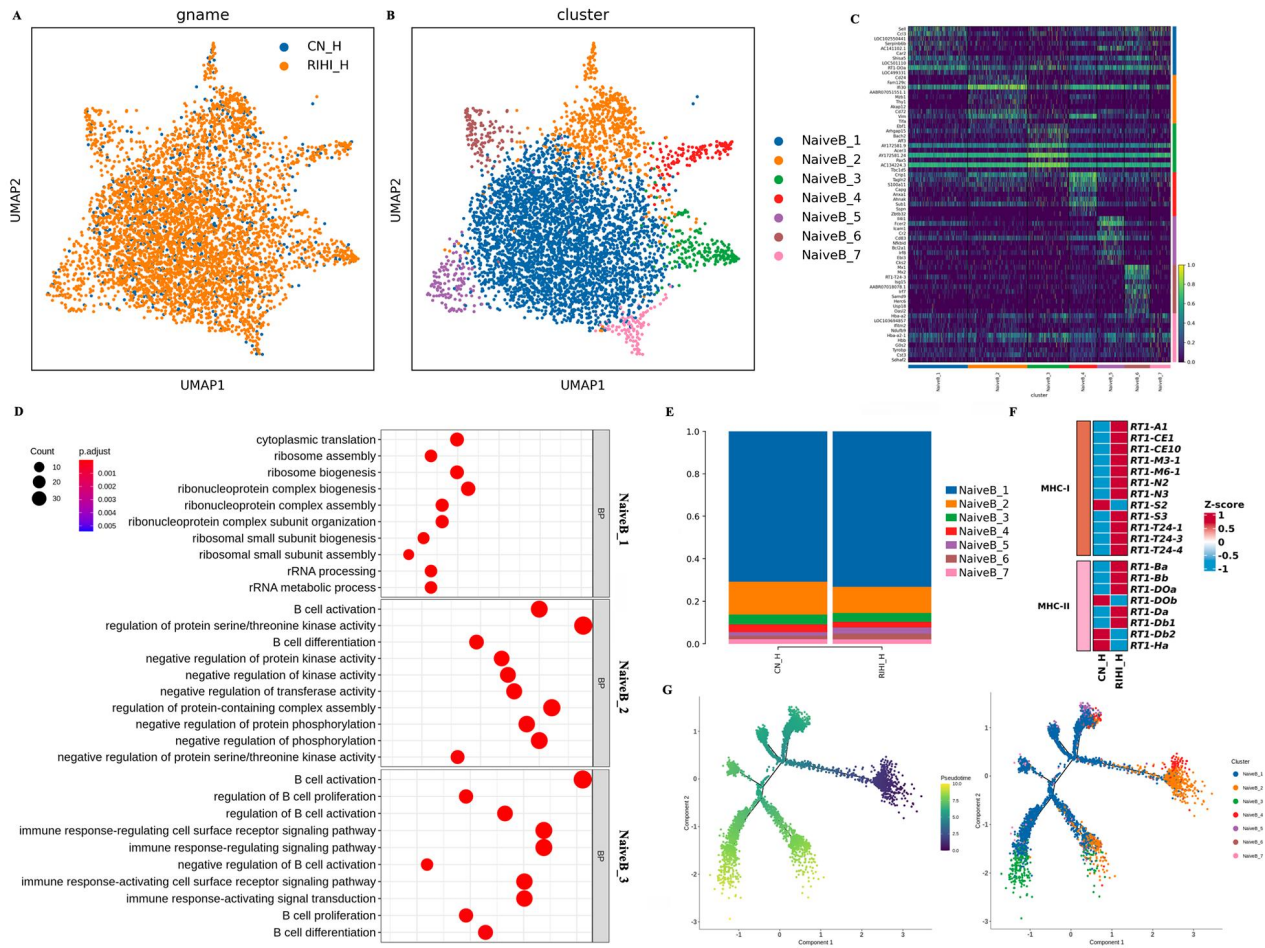
Single-cell landscape of B cells in RIHI and control rat. **(A,B)** UMAP for cell types of RIHI and control heart, including NaiveB1_7 (*n* = 5,003 cells); **(C)** Heatmap of canonical marker genes defining B-cell subsets; **(D)** The representative top 10 GO terms enriched in NaiveB1_3; **(E)** The relative proportion of each cell cluster between groups; **(F)** Heatmap showing the relative change in the expression level of signature genes of MHC-I and MHC-II in all B cells pre- and post-irradiation; **(G)** Pseudotime trajectory analysis of B cells. The color from heavy to light represents development from beginning to end.

B cells, traditionally viewed as mediators of humoral immunity, also serve as potent antigen-presenting cells within the cardiac immune microenvironment. We resolved 5,131 cardiac B cells into seven naïve subclusters (>98%) plus a minor plasma-cell fraction (Figures 1B,C). Subtype marker and GO profiles indicated: Cluster_1 (Sell, Ccl3, RT1-Doa) as the predominant tissue compartment with enrichment for ribosome/biogenesis programs; Cluster_2 (Cd24, Ifi30, Cd72) linked to protein regulation and B-cell activation; and Cluster_3 enriched for immune-response pathways (Figures 7A–D).

Following radiation, a mild expansion of Cluster_1 and a corresponding decrease in Cluster_2 were observed (Figure 7E), while the frequency of plasma B cells remained largely unchanged (Figure 1F). Importantly, irradiated B cells exhibited significant upregulation of genes involved in both MHC class I and class II antigen processing and presentation pathways, consistent with heightened antigen presentation capacity (Figure 7F).

Trajectory inference ordered B cells from Cluster_2 through a Cluster_1-dominated trunk and then into multiple branch endpoints. Along pseudotime, MHC processing modules (e.g., Spib, Cd79b, Ms4a1, Ifi30) transiently peaked, consistent with the radiation-induced augmentation of MHC-I/II pathways. Branches resolved into a migratory/inflammatory program (Ccl3, Sell), while plasma blast features were not sustained (no persistent Mzb1). Thus, irradiated B cells preferentially transition from naïve to antigen-presenting, trafficking-competent states rather than plasma-cell differentiation (Figure 7G, Supplementary Figures S4H,I).

## Discussion

RIHI remains a major late effect of thoracic radiotherapy, with incidences reported between 15.5% and 36% (28, 29). Most prior studies relied on bulk tissue and could not resolve how distinct cardiac and immune cell types cooperate to convert vascular damage into sustained inflammation (10). By single-cell profiling of irradiated hearts (38,941 single cells) and matched PBMCs (41,097 single cells) at 12 weeks, we delineate a multicellular cascade in which endothelial and stromal compartments undergo immunogenic reprogramming, build chemokine gradients, and recruit/shape myeloid and lymphoid responses, ultimately stabilizing a chronic antigen-presentation–inflammation loop.

A central finding is the coordinated immunization of the vascular-stromal interface. After irradiation, CapECs decline, whereas VECs and LECs expand and acquire immunoregulatory features: MHC-II and co-stimulatory molecules (Cd80, Cd86) are upregulated, accompanied by increased chemokines such as CCL21. This indicates a shift of the endothelium from a passive barrier to an APC-like node capable of activating T cells, in line with recent reports that endothelial cells can inducibly express MHC-II and trigger T-cell responses in inflammatory and immune settings (30). Future studies using co-culture systems, antigen-specific T-cell activation assays, and MHC-II blockade approaches will be required to determine whether these stromal populations directly contribute to adaptive immune activation during RIHI. Concurrently, in CapECs, upregulation of mitochondrial genes and ECM components was also noticed, suggesting impaired oxidative phosphorylation with matrix remodeling, consisting with radiation-induced mitochondrial damage, ROS imbalance, adhesion/thrombotic activation, and microvascular dysfunction (31–36). These findings extend the notion of “barrier disruption” to the establishment of an “antigen-presentation plus chemokine field”: the former provides signal strength while the latter supplies cellular flux.

Converging with the endothelium, cardiac fibroblasts exhibited a similar transformation. Rather than acting as passive scar donors, fibroblasts also upregulated MHC-II pathways and strengthened CXCL12–CXCR4 communication with ECs, T/NK cells, and macrophages, while displaying a ferroptosis-related adaptation. Ferroptosis is a regulated necrotic death driven by iron-catalyzed lipid peroxidation (37, 38). The maintenance of iron homeostasis is crucial for proper cardiac function, with mounting evidence suggesting that iron imbalance underpins various subtypes of cardiovascular disease (39, 40). In our work, we observed increased total iron content without amplification of lipid peroxidation, accompanied by enhanced antioxidant phenotypes, consistent with an anti-ferroptosis, cell preservation program that supports sustained antigen presentation and matrix remodeling (41, 42).

Downstream, innate immunity was amplified and diversified. Macrophages, as prominent intrinsic immune cells infiltrating the heart, perform diverse functions in heart injury, including the production of inflammatory cytokines and repair molecules, as well as the phagocytosis of cellular debris (43, 44). In our study, we meticulously characterized 990 cardiac macrophages into three heterogeneous subgroups. Following irradiation, macrophages underwent subpopulation reshaping, with Fcn^high^ clusters expanding and enhancing MHC-I antigen presentation potential, consistent with their role as CD8^+^ T-cell activation nodes (45, 46). In neutrophils, IL-1β^+^/NLRP3 programs and leukocyte-activation signatures are enriched, and KEGG points to links with Th1/Th17 differentiation and the PD-1/PD-L1 axis, indicating that neutrophils act not only as “first responders” but also as early instructors of adaptive immunity. Moreover, these innate programs fit within chemokine fields emanating from ECs and fibroblasts (e.g., CXCL12-CXCR4/ACKR3), providing spatial logic for myeloid recruitment and positioning.

Beyond the innate response, adaptive immune cells further consolidate this cascade. After myocardial infarction or infection, the immune system clears dead tissue, but it can also drive adverse remodeling and irreversible damage (47, 48). In our data, naïve T cells decreased while CD8^+^ effector T cells increased in cardiac tissue, with amplified cytotoxic programs yet features of partial exhaustion, consistent with a biphasic “activation-functional cost” state. Concordantly, Schlaak et al. reported increased T-cell infiltration after image-guided whole-heart RT, highlighting the multifaceted role of adaptive immunity in radiation-induced cardiac dysfunction (49). In PBMCs, we noted a modest rise in CD4 naïve T cells and a decrease in CD4^+^ Tregs, potentially reflecting a systemic response to cardiac immune activation. Cardiac T cells also displayed altered lipid metabolism with reduced FABP4 expression. Prior studies show that fatty-acid and cholesterol biosynthesis are critical for T-cell proliferation and differentiation (50), and that FABP4 integrates metabolic and inflammatory signaling to maintain T-cell fitness (51, 52). We therefore posit that FABP4-linked lipid metabolism contributes to T-cell dysfunction in RIHI. Together with fibroblast iron-antioxidant adaptation, these data outline a metabolism-immunity coupling vascular/stromal compartments preserve presentation and chemotaxis via anti-ferroptosis adaptations, while T cells operate under lipid-metabolic constraint and incur functional costs. B cells, although predominantly naïve, upregulated MHC-I/II pathways after irradiation and showed an increase in inflammatory Sell^+^/Ccl3^+^ subsets. Pseudotime analysis indicated progression toward terminal, antigen-processing competent states, supporting a role for B cells as auxiliary presenters and amplifiers of adaptive immunity. Acting with APC-like ECs and fibroblasts, this multi-origin antigen-presentation network provides architectural support for persistent immune infiltration.

Integrating these tiers yields a testable causal chain from radiation injury to chronic inflammation. MHC-II-positive endothelium and fibroblasts co-localize “antigen + chemokine” cues that recruit and prime leukocytes; Fcn^high^/MHC-I-leaning macrophages together with IL-1β^+^ neutrophils bridge innate inflammation to CD8^+^ effector programs; FABP4^low^ T cells deliver short, high-intensity cytotoxic bursts under metabolic constraint and drift toward dysfunction; and B cells, via MHC-I/II upregulation, amplify adaptive responses. This integrated framework also explains the heightened cardiotoxicity with radiotherapy plus immune checkpoint inhibitors (ICIs): RT increases antigen exposure and activates endothelial chemokine/presentation axes, while ICIs release peripheral brakes-together escalating myocardium-directed immune amplification (53, 54).

Building on this, we further outline actionable interventions that directly map to the inferred nodes: (1) temper non-classical APC functions in ECs/fibroblasts (e.g., MHC-II, CD80/CD86) to raise the activation threshold; (2) modulate fibroblast ferroptosis-resistance programs to reduce immunogenic signaling without provoking necrosis; and (3) restore T-cell lipid metabolism (e.g., FABP4-linked pathways) to preserve antitumor efficacy while mitigating off-target myocardial injury. These strategies may offer a tractable roadmap for precision cardio-oncology.

Although the present study provides a comprehensive single-cell characterization of RIHI in a controlled experimental model, validation in human samples will be important to determine the clinical relevance of the identified immune–stromal remodeling programs. Future studies incorporating peripheral blood and, when available, cardiac specimens from patients undergoing thoracic radiotherapy may help establish the translational significance of these findings.

## Conclusion

In summary, our integrated single-cell transcriptomic analysis of irradiated cardiac tissue and matched PBMCs delineates a coherent, three-layered framework of RIHI pathogenesis: vascular-stromal immunization and chemokine field formation; myeloid-driven antigen processing and inflammatory amplification; and the settlement of metabolically perturbed adaptive lymphocytes. This persistent MHC-II–dominated immune circuit integrates local tissue injury with systemic immune activation, reshaping the concept of RIHI from a single-endothelial injury model into a pan-cellular immune–metabolic disease.

## Data Availability

The raw data supporting the conclusions of this article will be made available by the authors, upon reasonable request.

## Supplementary material

Supplementary Figure 1 H&E staining of heart sections from control **(A)** and irradiated **(B)** rats at 12 weeks post-radiation, showing normal myocardial architecture in controls and increased interstitial infiltration and structural disruption in irradiated samples (scale bar = 120 μm); **(C)** Dot plot showing expression patterns of canonical marker genes across major cardiac cell populations.

Supplementary Figure 2 KEGG pathway enrichment analysis of differentially expressed genes in VEC **(A)** and LEC **(B)**; **(C)** Heatmap of pseudotime-ordered genes across endothelial subclusters. **(D)** Pseudotime expression plots of representative genes across endothelial subclusters.

Supplementary Figure 3 **(A,B)** Pseudotime trajectory analysis in fibroblast; **(C)** Heatmap of pseudotime-ordered genes across fibroblast states; **(D)** Pseudotime expression plots of representative genes in fibroblast.

Supplementary Figure 4 **(A–C)** KEGG pathway enrichment of differentially expressed genes across Neutrophil 1_3; **(D)** and **(E)** UMAP for cell types of T and NK cells in PBMC; **(F)** Heatmap of canonical marker genes for T and NK cells annotation in PBMC; **(G)** Proportional distribution of T/NK subsets before and after irradiation in PBMC; **(H)** Pseudotime trajectory analysis showing differentiation dynamics and lineage progression among B cell subsets; **(I)** Heatmap of pseudotime-ordered genes, illustrating transcriptional changes during B cell activation and state transitions.
