# Supplementary figures and images for: Immune and stromal remodeling underlies radiation-induced heart injury: insights from single-cell transcriptomics
Source: Front Cardiovasc Med. 2026 Jul 2;13:1836858. doi: 10.3389/fcvm.2026.1836858 (PMC13372902; doi:10.3389/fcvm.2026.1836858)

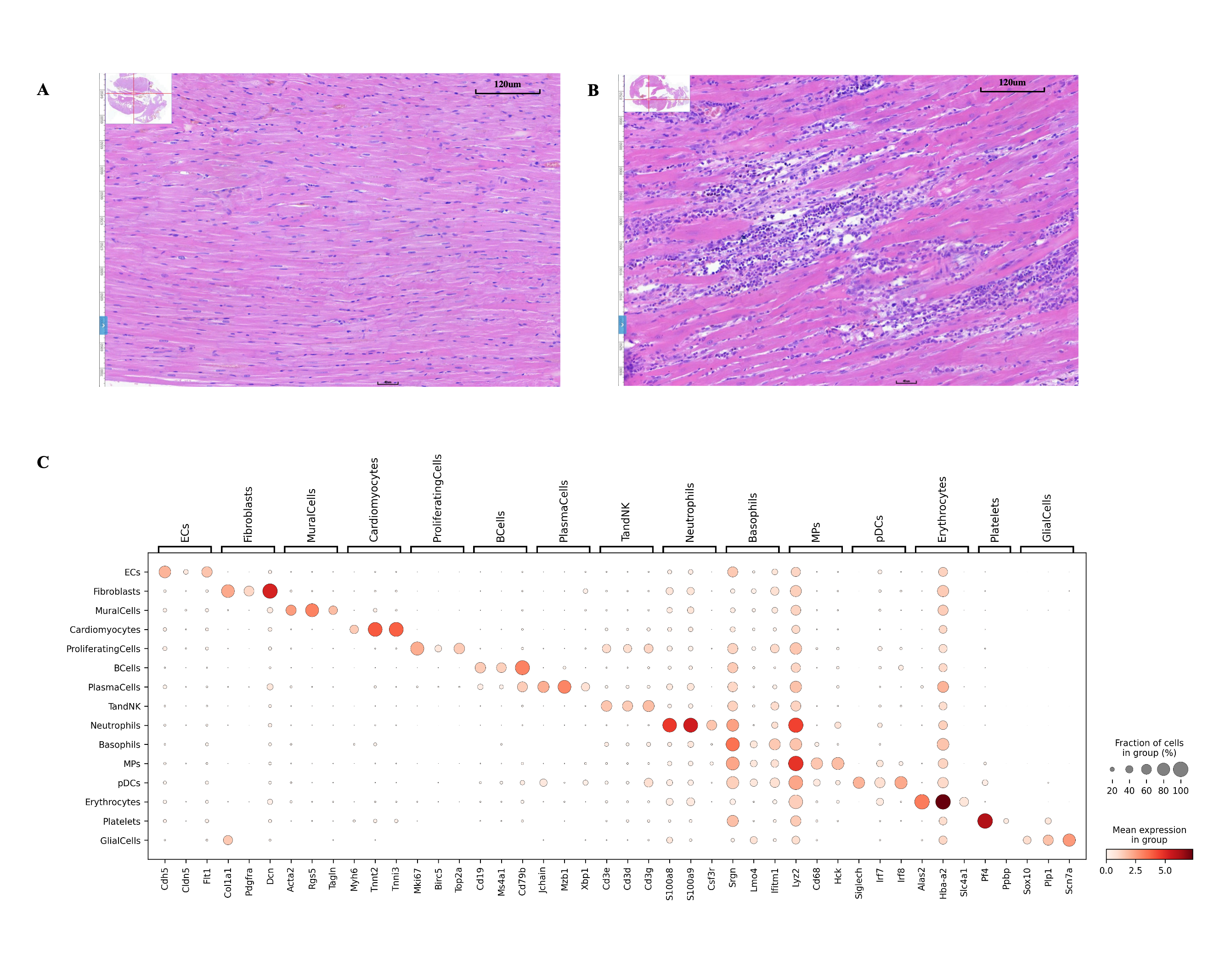

Supplement: Supplementary Figure 1 — H&E staining of heart sections from control (A) and irradiated (B) rats at 12 weeks post-radiation, showing normal myocardial architecture in controls and increased interstitial infiltration and structural disruption in irradiated samples (scale bar = 120 μm); (C) Dot plot showing expression patterns of canonical marker genes across major cardiac cell populations. [file Image1.tiff]

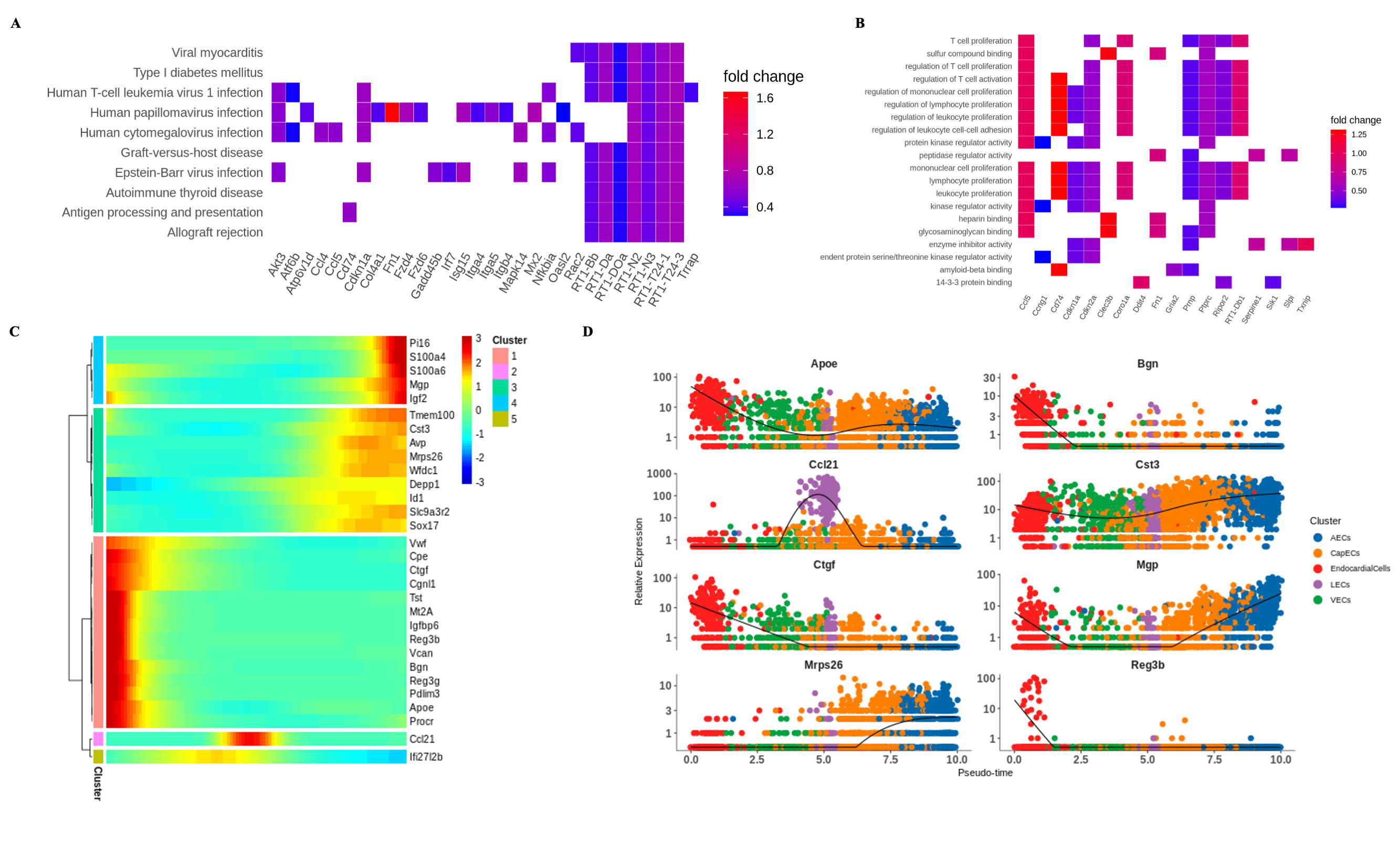

Supplement: Supplementary Figure 2 — KEGG pathway enrichment analysis of differentially expressed genes in VEC (A) and LEC (B); (C) Heatmap of pseudotime-ordered genes across endothelial subclusters. (D) Pseudotime expression plots of representative genes across endothelial subclusters. [file Image2.tiff]

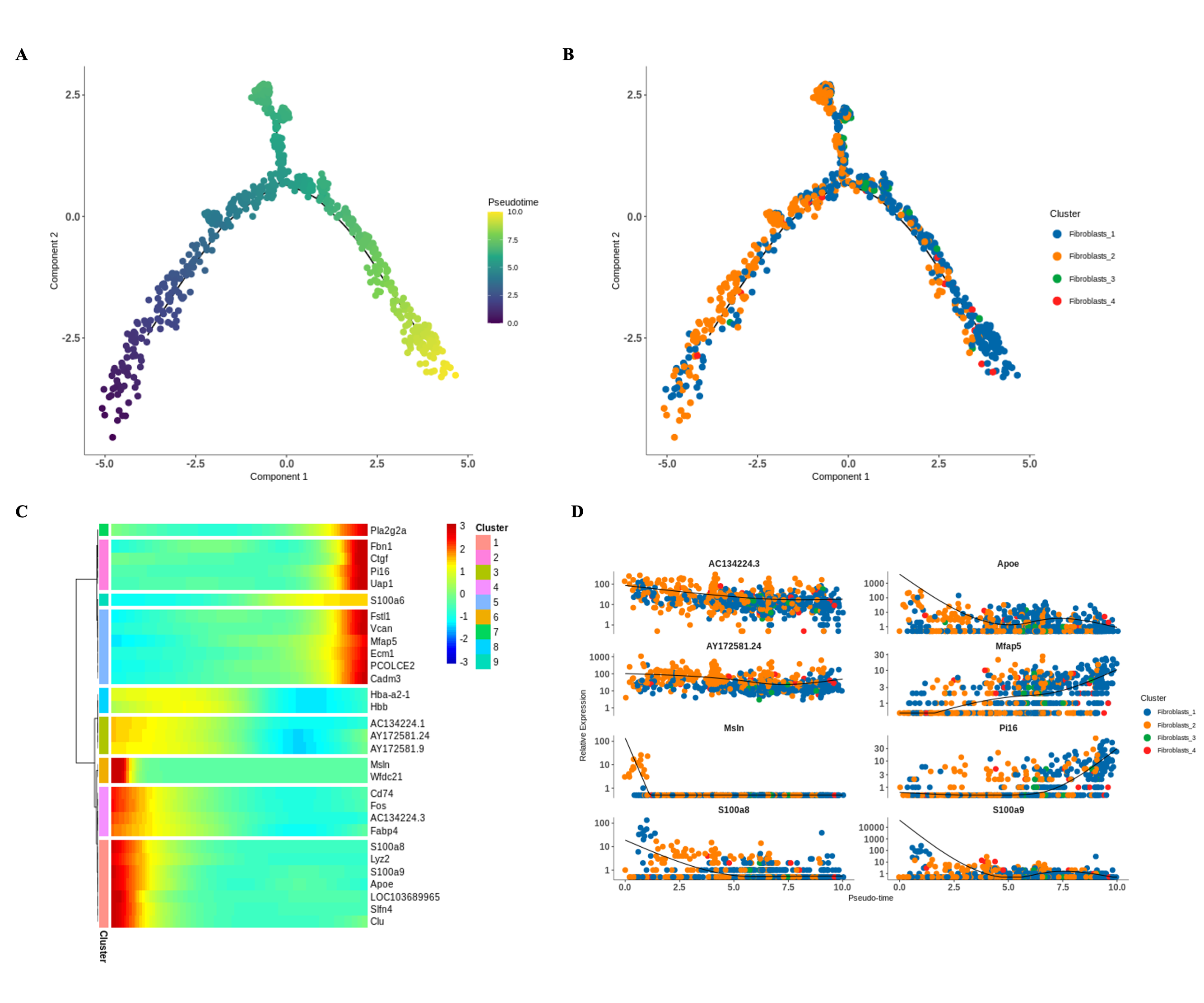

Supplement: Supplementary Figure 3 — (A,B) Pseudotime trajectory analysis in fibroblast; (C) Heatmap of pseudotime-ordered genes across fibroblast states; (D) Pseudotime expression plots of representative genes in fibroblast. [file Image3.tiff]

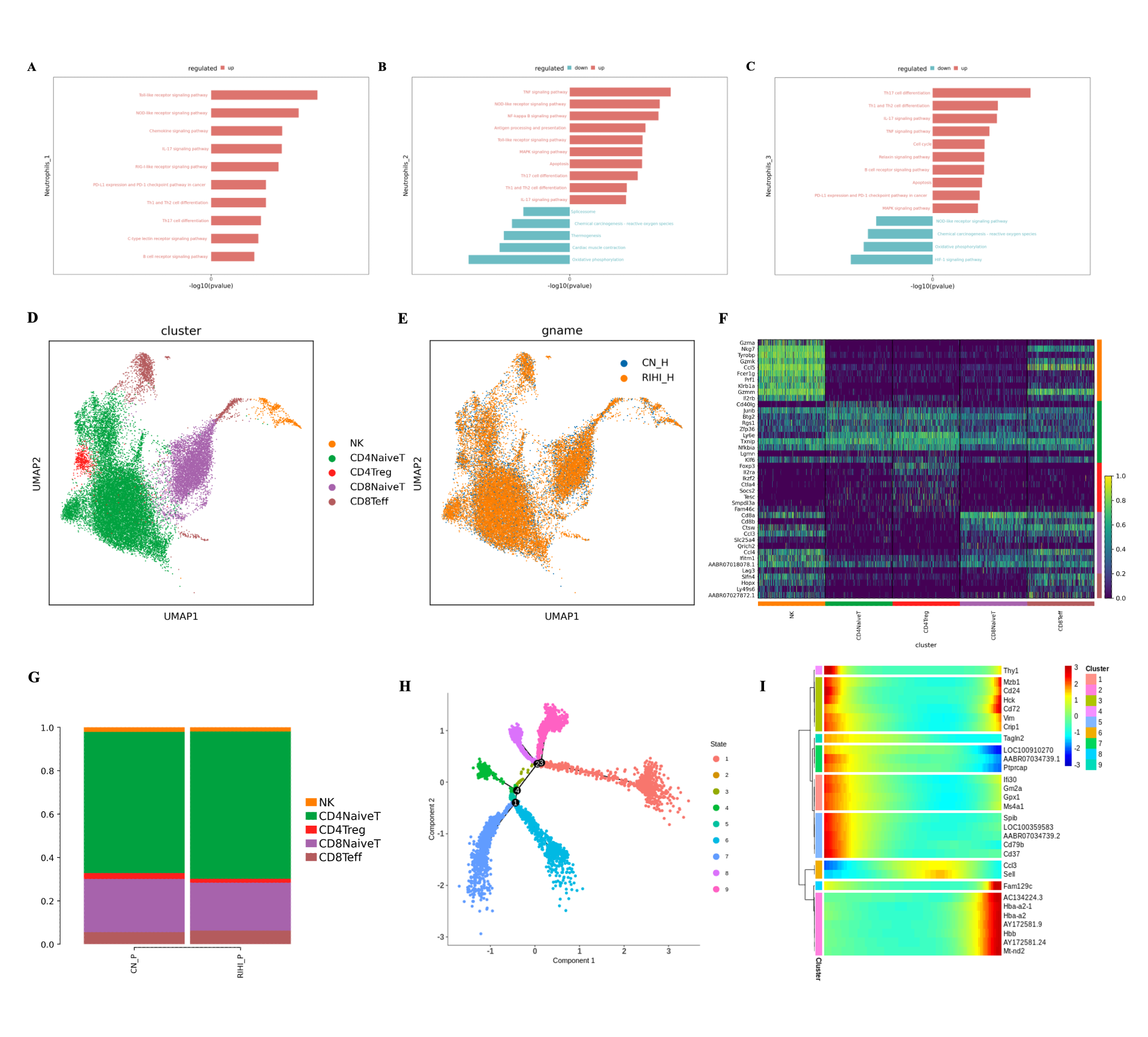

Supplement: Supplementary Figure 4 — (A–C) KEGG pathway enrichment of differentially expressed genes across Neutrophil 1_3; (D) and (E) UMAP for cell types of T and NK cells in PBMC; (F) Heatmap of canonical marker genes for T and NK cells annotation in PBMC; (G) Proportional distribution of T/NK subsets before and after irradiation in PBMC; (H) Pseudotime trajectory analysis showing differentiation dynamics and lineage progression among B cell subsets; (I) Heatmap of pseudotime-ordered genes, illustrating transcriptional changes during B cell activation and state transitions. [file Image4.tiff]
