# Supplementary material for: Immune and stromal remodeling underlies radiation-induced heart injury: insights from single-cell transcriptomics
Source: Front Cardiovasc Med. 2026 Jul 2;13:1836858. doi: 10.3389/fcvm.2026.1836858 (PMC13372902; doi:10.3389/fcvm.2026.1836858)
Supplement: Supplementary file 5 [file Datasheet1.docx]

| **Supplementary Table1: Proportion of each identified cell type in the heart** | | | | |
| --- | --- | --- | --- | --- |
| **Cell types** | **Number**  **CN_H** | **Number**  **RIHI_H** | **Proportion CN_H** | **Proportion**  **RIHI_H** |
| Glial Cells | 24 | 15 | 0.00212615 | 0.00054244 |
| ECs | 4672 | 6538 | 0.41389086 | 0.23643004 |
| Fibroblasts | 753 | 220 | 0.06670801 | 0.00795574 |
| Mural Cells | 499 | 198 | 0.04420624 | 0.00716016 |
| Cardiomyocytes | 215 | 150 | 0.01904678 | 0.00542437 |
| Proliferating Cells | 50 | 222 | 0.00442948 | 0.00802806 |
| BCells | 662 | 4408 | 0.05864635 | 0.15940404 |
| Plasma Cells | 31 | 67 | 0.00274628 | 0.00242288 |
| T and NK | 2119 | 8209 | 0.18772147 | 0.29685748 |
| Neutrophils | 1003 | 1211 | 0.08885542 | 0.04379272 |
| Basophils | 3 | 13 | 0.00026577 | 0.00047011 |
| MPs | 1025 | 5864 | 0.09080439 | 0.21205656 |
| pDCs | 7 | 45 | 0.00062013 | 0.00162731 |
| Erythrocytes | 208 | 451 | 0.01842665 | 0.01630926 |
| Platelets | 17 | 42 | 0.00150602 | 0.00151882 |

| **Supplementary Table2: Proportion of each identified cell type in PBMC** | | | | |
| --- | --- | --- | --- | --- |
| **Cell types** | **Number**  **CN_H** | **Number**  **RIHI_H** | **Proportion**  **CN_H** | **Proportion**  **RIHI_H** |
| B Cells | 2086 | 7939 | 0.1864331 | 0.26544737 |
| Plasma Cells | 24 | 80 | 0.00214496 | 0.00267487 |
| T and NK | 7494 | 18203 | 0.66976495 | 0.60863314 |
| Neutrophils | 201 | 1098 | 0.01796407 | 0.03671259 |
| Basophils | 25 | 86 | 0.00223434 | 0.00287548 |
| MPs | 1254 | 1691 | 0.11207436 | 0.05654006 |
| pDCs | 11 | 24 | 0.00098311 | 0.00080246 |
| Erythrocytes | 21 | 421 | 0.00187684 | 0.0140765 |
| Platelets | 73 | 366 | 0.00652426 | 0.01223753 |
